# Efficacy and safety of neoadjuvant immunotherapy protocols and cycles for non-small cell lung cancer: a systematic review and meta-analysis

**DOI:** 10.3389/fonc.2024.1276549

**Published:** 2024-01-16

**Authors:** Huaiyong Wang, Song Liang, Yue Yu, Yun Han

**Affiliations:** Department of Thoracic Surgery, Shengjing Hospital of China Medical University, Shenyang, Liaoning, China

**Keywords:** lung cancer, preoperative immunotherapy, treatment regimens, neoadjuvant cycle, major pathologic response, meta-analysis

## Abstract

**Objectives:**

This study evaluated the use of different neoadjuvant immunotherapy cycles and regimens for non-small cell lung cancer.

**Materials and methods:**

Databases were searched for articles published up until December 2023. Data on the major pathologic response (MPR), complete pathologic response (pCR), radiological response, treatment-related adverse events (TRAEs), serious adverse events (SAEs), surgical resection, surgical complications, R0 resection, and conversion to thoracotomy were collected. A subgroup analysis was performed according to the treatment regimens and cycles. Stata/MP software was used for statistical analyses.

**Results:**

In total, 2430 individuals were assessed from 44 studies. Compared with those following neoadjuvant immunotherapy alone (MPR/pCR/TRAEs/SAEs: ES=0.26/0.07/0.43/0.08, 95% CI: 0.18-0.34/0.04-0.10/0.28-0.58/0.04-0.14), the MPR and pCR rates, incidence of TRAEs and SAEs following neoadjuvant chemoimmunotherapy increased significantly (MPR/pCR/TRAEs/SAEs: ES=0.55/0.34/0.81/0.22, 95% CI: 0.48-0.63/0.28-0.41/0.69-0.90/0.13-0.33, P=0.001/0.002/0.009/0.034). No significant differences were found in the surgical resection, surgical complications, R0 resection, or conversion to thoracotomy. In the chemoimmunotherapy group, no statistically significant differences were found in the MPR and pCR rates, incidence of TRAEs and SAEs in the two-cycle, three-cycle and four-cycle groups (MPR/pCR/TRAEs/SAEs: ES=0.50;0.70;0.36/0.32;0.49;0.18/0.95;0.85;0.95/0.34;0.27;0.37, P=0.255/0.215/0.253/0.848). In the ICIs group, there was little change in the MPR and pCR rates, incidence of TRAEs and SAEs in the two-cycle group compared to the three-cycle group. (MPR/pCR/TRAEs/SAEs: ES=0.28;0.20/0.06;0.08/0.45;0.35/0.10;0.02, P=0.696/0.993/0.436/0.638). The neoadjuvant treatment cycle had no significant effect on surgical resection, surgical complications, R0 resection, or conversion to thoracotomy in both regimens.

**Conclusion:**

Neoadjuvant chemoimmunotherapy significantly increased the rate of tumor pathological remission compared to neoadjuvant immunotherapy alone but also increased the incidence of TRAEs and SAEs. The efficacy and safety of neoadjuvant chemoimmunotherapy are found to be favorable when administered for a duration of three cycles, in comparison to both two and four cycles.

**Systematic review registration:**

https://www.crd.york.ac.uk/PROSPERO/#recordDetails, identifier CRD42023407415.

## 1 Introduction

Lung cancer is second only to breast cancer in terms of incidence worldwide and has the highest mortality rate among malignant tumors (1). From 2010 to 2019, the number of new tracheal, bronchial, and lung cancer cases increased by 23.3% (2). Therefore, effective interventions for lung cancer that prolong patient survival are needed. Radical surgery combined with neoadjuvant and adjuvant therapies, when necessary, has become the mainstay of treatment for non-metastatic lung cancer.

In recent years, programmed cell death protein 1 and programmed death-ligand 1 inhibitors have demonstrated unique therapeutic benefits in the neoadjuvant treatment of melanoma, hepatocellular carcinoma, and other tumors (3, 4). In 2018, CheckMate159 (5, 6) reported a 45% major pathologic response (MPR) rate and 24% incidence of treatment-related adverse events (TRAEs) following neoadjuvant immunotherapy in non-small cell lung cancer (NSCLC), which confirmed the feasibility and safety of the treatment. This led to a series of clinical studies on preoperative immunotherapy and immunotherapy combined with chemotherapy or radiotherapy. However, the results of these studies have been inconsistent. It cannot be excluded that the differences are related to indicators such as the neoadjuvant treatment regimen, cycle, or type of immune checkpoint inhibitors (ICIs). Previous meta-analyses have confirmed that different ICIs have no significant impact on the safety and feasibility of treatment (7). Therefore, we conducted a meta-analysis of the different neoadjuvant immunotherapy regimens and cycles.

## 2 Materials and methods

This study followed the Preferred Reporting Items for Systematic Reviews and Meta-Analyses (PRISMA statement) and was registered in the International Prospective Register of Systematic Reviews (PROSPERO CRD42023407415).

### 2.1 Search strategy

We retrieved relevant studies on neoadjuvant immunotherapy for lung cancer by searching seven databases, including PubMed, Embase, Cochrane Library, Ovid, Scopus, ProQuest, and Web of Science, published through December 2023. The search terms were as follows: (“Carcinoma, Non-Small-Cell Lung” OR “Lung Carcinoma, Non-Small-Cell” OR “NSCLC”) AND (“Neoadjuvant Therapy” OR “Neoadjuvant Treatment” OR “Neoadjuvant Radiotherapy” OR “Neoadjuvant Chemotherapy” OR “Neoadjuvant Systemic Therapy”) AND (“Immunotherapy” OR “Immunotherapies”).

The inclusion criteria were as follows: 1. Patients with pathologically confirmed stage I–IV lung cancer and the possibility of surgical resection; 2. preoperative application of neoadjuvant immunotherapy or immunotherapy combined with other treatments, such as chemotherapy and radiotherapy; and 3. complete patient characteristics and inclusion of important outcome indicators, such as pathological response, radiological response, TRAEs, and surgery-related data. The exclusion criteria were as follows: 1. The primary endpoint of the study was not related to the efficacy or safety of the neoadjuvant therapy; 2. studies that have not been completed; 3. duplicate publications and data; 4. sample size <10; 5. reviews, conference abstracts, case reports, animal studies, and cytological studies; 6. non-English literature. Two investigators independently searched and screened the articles separately, resolved differences through discussions, and determined the final search results.

### 2.2 Data extraction

Two researchers read the original and **Supplementary Materials** of the included publications and extracted the following relevant data: 1. Article author(s), year of publication, National Clinical Trial (NCT) number, sample size, and primary endpoint; 2. patient age, sex ratio, smoking ratio, pathological type, tumor stage, neoadjuvant treatment regimen, and cycles; and 3. pathological response (complete pathologic response [pCR], MPR), radiological response, the incidence of TRAE, and grading; and 4. surgical resection rate, surgical delay rate, the incidence of surgical complications, surgical style, and R0 resection rate (**Table 1**).

**Table 1:** Summary of studies on neoadjuvant immunotherapy for NSCLC.

Study	NCT Number	Clinical trial	Study type	Neoadjuvant treatment regimen	Cycles	Male n[%]	Age	Smoking history n(%)	Clinical stage	MPR	pCR	CR+PR	TRAEs	SAEs	Surgical resection	Surgical complications	R0 resection	Conversion to thoracotomy
Bott, 2019 (5) Forde, 2018 (6)	NCT02259621	CheckMate159	Cohort study	Nivolumab	2	10 (48.0%)	66.9	18 (86.0%)	IA-IIIA	45.0%	15.0%	9.5%	23.8%	4.8%	95.2%	50.0%	NR	35.0%
Forde, 2022 (8)	NCT02998528	CheckMate816	RCT	Nivolumab+CT	3	128 (71.5%)	64.0	160 (89.4%)	IB-IV	36.9%	24.0%	63.7%	81.0%	33.0%	83.2%	41.6%	83.2%	11.4%
Heymach, 2023 (9)	NCT03800134	AEGEAN	RCT	Durvalumab+CT	4	252 (68.9%)	65.0	315 (86.1%)	IIA-IIIB	33.3%	17.2%	56.3%	86.8%	32.4%	77.6%	NR	94.7%	NR
Wakelee, 2023 (10)	NCT03425643	KEYNOTE671	RCT	Pembrolizumab+CT	4	279 (70.3%)	63.0	343 (86.4%)	IIA-IIIB	30.2%	18.1%	NR	95.7%	40.7%	82.1%	NR	92.0%	NR
Sepesi, 2022 (11) Cascone, 2021 (12)	NCT03158129	NEOSTAR	RCT	Nivolumab (+ipilimumab)	3	15 (65.0%)	66.1	18 (78.0%)	IA-IIIA	23.8%	9.5%	21.7%	NR	13.0%	91.3%	38.1%	NR	4.8%
Chaft, 2022 (13) Rusch, 2023 (14)	NCT02927301	LCMC3	Cohort study	Atezolizumab	2	88 (49.0%)	65.0	163 (90.0%)	IB-IIIB	20.3%	5.6%	6.1%	60.8%	11.0%	87.8%	NR	91.2%	9.4%
Provencio, 2020 (15) Provencio, 2022 (16)	NCT03081689	NADIM	Cohort study	Nivolumab+CT	3	34 (74.0%)	63.0	46 (100%)	IIB-IIIA	82.9%	63.4%	76.1%	93.5%	34.8%	89.1%	29.3%	100.0%	9.8%
Shu, 2020 (17)	NCT02716038	Columbia/MGH	Cohort study	Atezolizumab+CT	4	15 (50.0%)	67.0	30 (100.0%)	IIB-IIIB	65.4%	38.5%	63.3%	NR	NR	96.7%	NR	100.0%	NR
Rothschild, 2021 (18)	NCT02572843	SAKK 16/14	Cohort study	Durvalumab+CT	2	35 (52.0%)	61.0	64 (95.0%)	IIIA(N2)	61.8%	18.2%	53.7%	100.0%	88.1%	82.1%	NR	92.7%	NR
F Zhang, 2022 (19) S Gao, 2020 (20)	ChiCTR-OIC -17013726	NR	Cohort study	Sintilimab	2	33 (82.5%)	59.8	32 (80.0%)	IA-IIIB	40.5%	16.2%	20.0%	52.5%	10.0%	97.5%	10.3%	NR	0
Wislez, 2022 (21)	NCT03030131	IFCT-1601 IONESCO	Cohort study	Durvalumab	3	31 (67.4%)	60.9	45 (97.8%)	IB-IIIA	18.6%	7.0%	8.7%	34.8%	0	93.5%	32.6%	NR	NR
ZR Zhao,2021 (22)	NCT04304248	NR	Cohort study	Toripalimab+CT	3	27 (81.8%)	61.0	NR	IIIA-IIIB	66.7%	50.0%	87.9%	NR	NR	90.9%	NR	96.7%	3.3%
J Shen, 2023 (23)	NR	NR	Cohort study	Pembrolizumab/Nivolumab/Stintilimab (+CT)	1-3	26 (83.9%)	61.0	19 (61.3%)	IB-IIIB	46.4%	21.5%	71.0%	64.5%	NR	90.3%	NR	96.4%	NR
Jiang, 2021 (24)	NR	NR	Cohort study	Pembrolizumab/Nivolumab+CT	2–4	29 (93.5%)	61.0	7 (22.6%)	IIA-IIIB	38.7%	9.7%	77.4%	NR	NR	100.0%	58.1%	77.4%	3.2%
Lee, 2022 (25) Altorki, 2021 (26)	NR	NR	RCT	Durvalumab	2	16 (53.0%)	71.0	24 (80.0%)	IA-IIIA	7.7%	0.0%	3.3%	NR	16.7%	86.7%	NR	NR	0
Tong, 2021 (27)	NR	TOP1501	Cohort study	Pembrolizumab	2	16 (53.0%)	72.0	26 (86.7%)	IB-IIIIA	28.0%	12.0%	NR	NR	3.3%	83.3%	48.0%	NR	20.0%
Eichhorn, 2021 (28)	NCT03197467	NEOMUN	Cohort study	Pembrolizumab	2	7 (46.7%)	59.8	NR	IIB-IIIB	26.7%	13.3%	26.7%	33.3%	20.0%	100.0%	6.7%	NR	NR
Wu, 2022 (29)	NR	NR	Cohort study	Pembrolizumab/Nivolumab+CT	2–4	72 (95.0%)	62.0	51 (67.0%)	IB-IIIB	64.5%	36.8%	75.0%	NR	NR	100.0%	NR	100.0%	2.6%
Y Gao, 2022 (30)	ChiCTR2200057840	NR	Cohort study	Nivolumab/Camrelizumab/Toripalimab/Tislelizumab/Sintilimab/Pembrolizumab+CT	3	33 (75.0%)	61.5	33 (75.0%)	IIIA-IIIB	81.8%	59.1%	NR	81.8%	18.2%	100.0%	11.4%	100.0%	4.5%
Hu, 2021 (31)	NR	NR	Cohort study	Pembrolizumab/Tislelizumab/Sintilimab/Toripalimab+CT	2–4	18 (90.0%)	56.0	17 (85.0%)	IB-IIIB	40.0%	25.0%	75.0%	NR	0	100.0%	35.0%	100.0%	0
B Zhang, 2022 (32)	NR	NR	Cohort study	Pembrolizumab/Sintilizumab/Toripalimab/Camrelizumab/Nivolizumab/Tislelizumab	1-5	127 (96.9%)	59.3	120 (91.6%)	IB-IIIB	53.4%	NR	78.6%	NR	NR	100.0%	21.4%	95.4%	32.1%
Faehling, 2022 (33)	NR	KOMPASSneoOP	Cohort study	Pembrolizumab/Nivolumab+CT	NR	30 (51.0%)	63.6	53 (95.0%)	IIB-IVB	67.8%	52.5%	84.7%	NR	NR	100.0%	NR	94.9%	NR
T Chen, 2021 (34)	NR	NR	Cohort study	Nivolumab/Pembrolizumab+CT	2/4	9 (75.0%)	61.0	7(58.3%)	IIIA-IIIB	33.3%	41.7%	50.0%	33.3%	NR	100.0%	25.0%	100.0%	NR
P Zhang, 2022 (35)	ChiCTR1900023758	NR	Cohort study	Sintilimab+CT	2–4	44 (88.0%)	64.8	38 (76.0%)	IIIA	43.3%	20.0%	46.0%	90.0%	8.0%	60.0%	3.3%	100.0%	NR
Yang, 2017 (36)	NCT01820754	TOP1201	Cohort study	Ipilimumab+CT	2	12 (50.0%)	65.0	23 (96.0%)	IIA-IIIA	NR	15.4%	58.3%	NR	45.8%	54.2%	NR	100.0%	23.1%
Y Chen, 2021 (37)	NR	NR	Cohort study	Pembrolizumab+CT	2	29 (83.0%)	62.1	27 (77.1%)	IIIA-IIIB	22.9%	51.4%	48.6%	NR	2.9%	100.0%	NR	100.0%	NR
Duan, 2021 (38)	NR	NR	Cohort study	Sintilimab/Nivolumab/Pembrolizumab+CT	3–4	22 (95.7%)	61.8	22 (95.7%)	IIA-IIIB	50.0%	30.0%	73.9%	NR	NR	87.0%	NR	95.0%	10.0%
Tfayli, 2020 (39)	NCT03480230	NR	Cohort study	Avelumab+CT	4	7 (46.7%)	65.0	11 (73.3%)	IB-IIIA	27.3%	9.1%	26.7%	NR	26.7%	NR	NR	NR	NR
Sun, 2023 (40)	NCT04326153	NR	Cohort study	Sintilimab+CT	2–3	18 (90.0%)	56.9	18 (90.0%)	IIIA-IIIB	62.5%	31.3%	75.0%	70.0%	35.0%	80.0%	NR	100.0%	0
G Zhao, 2023 (41)	NR	NR	Cohort study	Pembrolizumab+CT	2-3	22 (88.0%)	65.0	9(36.0%)	IIB-IIIB	61.9%	28.6%	80.0%	NR	NR	84.0%	NR	100.0%	NR
Huang, 2021 (42)	NR	NR	Cohort study	Nivolumab	2	16 (64.0%)	62.9	15 (60.0%)	IIIA	37.5%	4.2%	32.0%	NR	12.0%	96.0%	12.5%	NR	0
Ma, 2022 (43)	NR	NR	Cohort study	Pembrolizumab+CT	3–5	27 (77.1%)	63.0	29 (82.9%)	IIB-IIIC	77.1%	48.6%	80.0%	17.1%	NR	100.0%	8.6%	NR	NR
Wang, 2021 (44)	NR	NR	Cohort study	Nivolumab/Pembrolizumab+CT	2	66 (91.7%)	62.2	60 (83.3%)	IIIA	NR	29.2%	94.4%	NR	NR	100.0%	NR	NR	NR
Zhai, 2022 (45)	NR	NR	Cohort study	Nivolumab+CT	3	26 (56.5%)	63.0	43 (93.5%)	IIIA-IIIB	71.1%	53.3%	60.9%	NR	19.6%	97.8%	NR	95.6%	0
Hong, 2021 (46)	NR	NR	Cohort study	Pembrolizumab/Sintilimab/Camrelizumab+CT	2–4	23 (92.0%)	62.0	179 (68.0%)	IIA-IIIC	52.0%	32.0%	88.0%	NR	0	100.0%	52.0%	100.0%	8.0%
Shen, 2021 (47)	NR	NR	Cohort study	Pembrolizumab+CT	2	35 (94.6%)	62.8	31 (83.8%)	IIB-IIIB	64.9%	45.9%	86.5%	70.3%	10.8%	100.0%	NR	100.0%	0
Yao, 2022 (48)	NR	NR	Cohort study	Camrelizumab/Durvalumab+CT	2–3	10 (90.9%)	67.7	10 (90.9%)	IIIA-IIIB	81.8%	72.7%	72.7%	72.7%	0	100.0%	27.3%	100.0%	9.1%
Liu, 2022 (49)	NR	NR	Cohort study	Pembrolizumab/Sintilimab/Camrelizumab+CT	2–5	66 (83.5%)	NR	66 (83.5%)	IB-IIIB	53.2%	NR	70.9%	NR	NR	100.0%	NR	100.0%	NR

CT, Chemotherapy; NR, Not reported.

### 2.3 Data analysis

This study used Stata/MP 17.0 software for the data analysis. The extent of data heterogeneity was determined using I^2^ and Q tests. A random effects model was used if the homogeneity test results were significant; otherwise, a fixed effects model was used. The pooled effect sizes (ES) were expressed as odds ratios (ORs) and 95% confidence intervals (CIs). Meta-regression was used to determine the differences among different neoadjuvant therapies. Publication bias was evaluated using funnel plots and Egger’s test, and differences were considered significant when *P* < 0.05. The stability of the results was evaluated using a sensitivity analysis.

### 2.4 Quality evaluation

**Figure 1:**
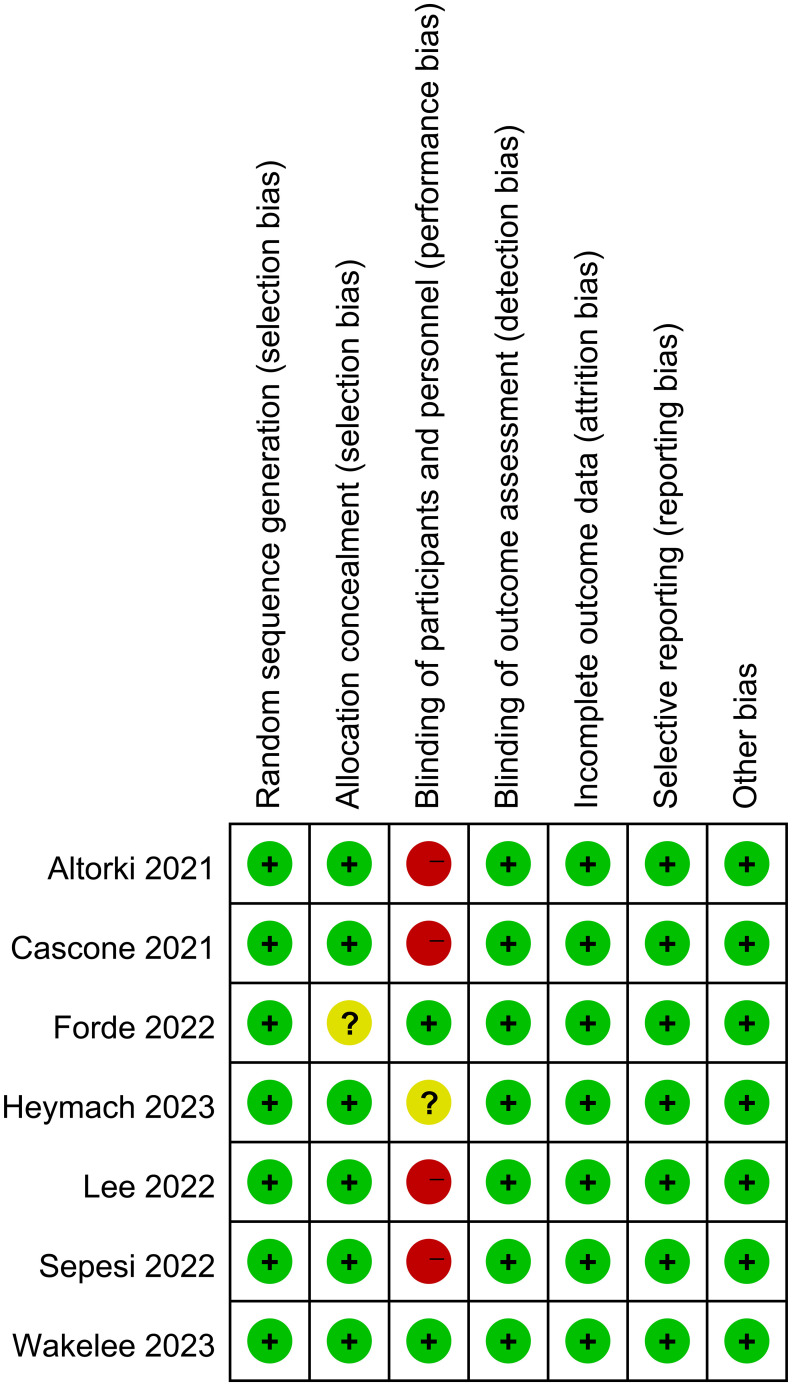
RCT literature evaluation.

The Cochrane Collaboration’s Risk of Bias tool was used to evaluate the quality of randomized controlled trials (RCTs) (**Figure 1**). For single-arm and cohort studies, the MINORS scale was used for evaluation (**Supplementary Table 1**).

## 3 Results

### 3.1 Search results

**Figure 2:**
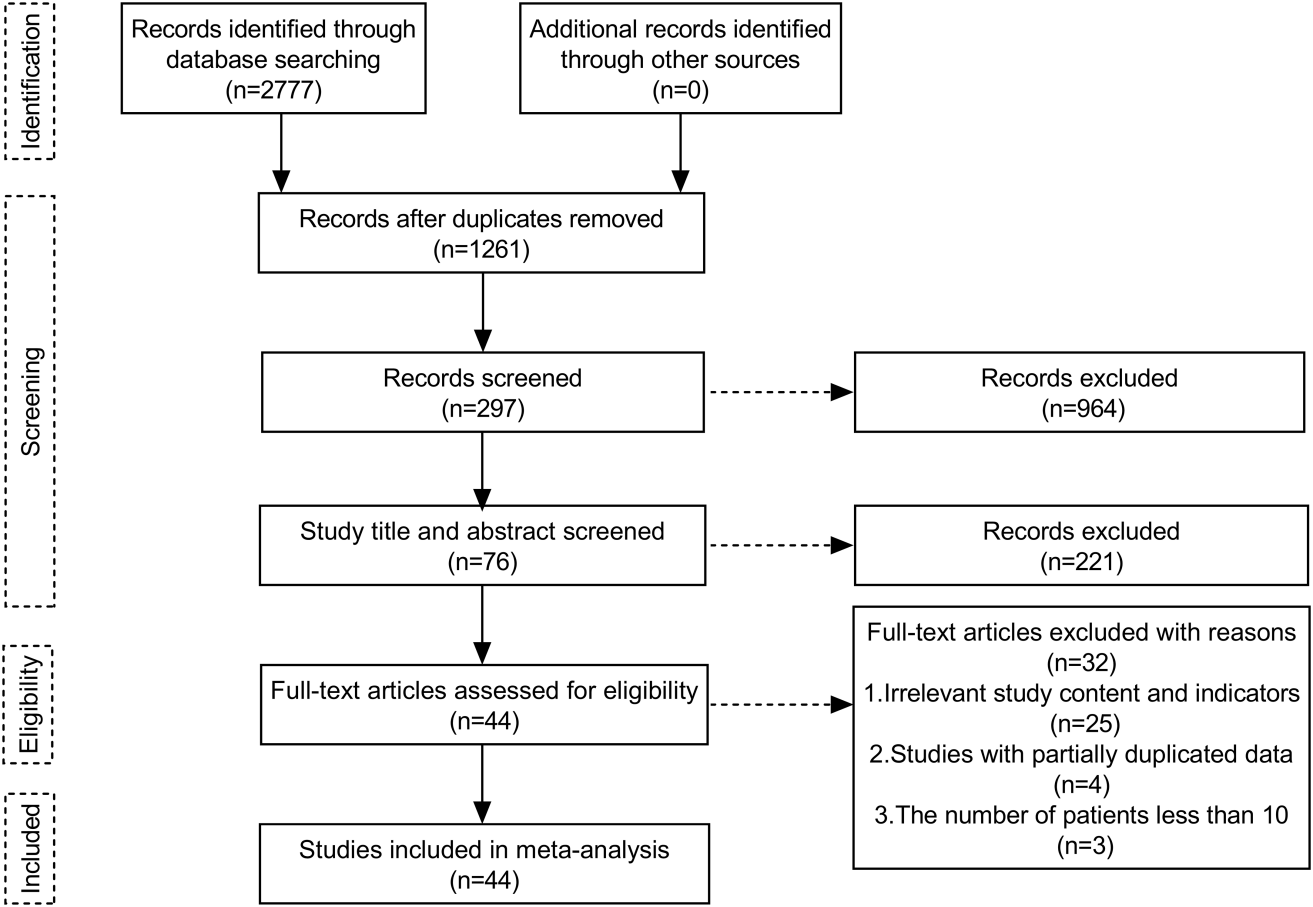
Flow chart of the meta-analysis search strategy.

A total of 2777 publications were retrieved. After removing duplications, 1261 publications remained. After reading records in the literature, abstracts, titles, and full texts, 44 papers were screened (**Figure 2**), of which 7 were RCT studies (Forde et al., 2022 (8), Heymach et al., 2023 (9), Wakelee et al., 2023 (10), Sepesi et al., 2022 (11), Cascone et al., 2021 (12), Lee et al., 2022 (25), Altorki et al., 2021 (26)). Only data from groups treated with immunotherapy alone or neoadjuvant chemoimmunotherapy were analyzed.

### 3.2 Primary outcomes

#### 3.2.1 Effectiveness of treatment

**Figure 3:**
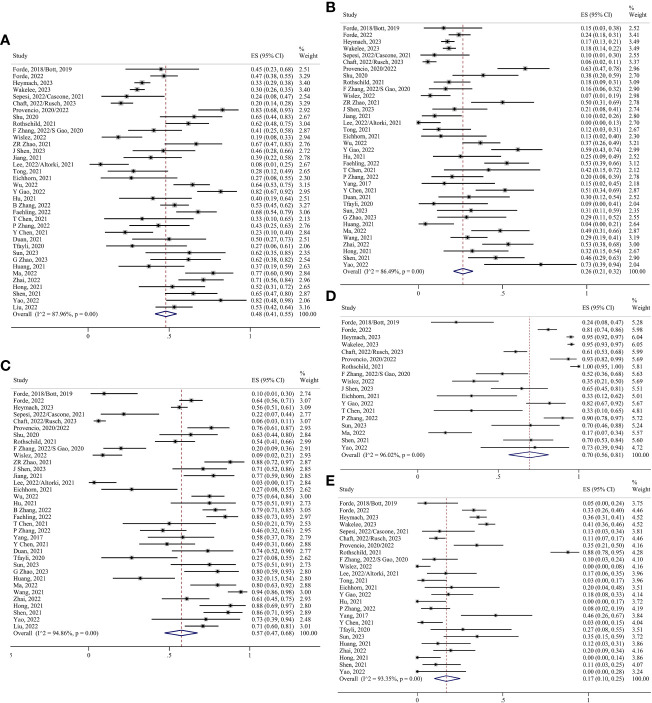
Forest plots. **(A)** MPR. **(B)** pCR. **(C)** Radiological response. **(D)** TRAEs. **(E)** SAEs.

MPR was defined as there being less than 10% of residual viable tumor cells in the primary tumor, and pCR was defined as there being no residual viable tumor cells in the primary tumor. Thirty-six studies have evaluated MPR (5, 6, 8–35, 37–43, 45–49), with MPR rates ranging from 8–83%. pCR rates of 0–73% have also been reported in 36 studies (5, 6, 8–31, 33–48). Because of the high heterogeneity, the random effects model analysis suggested that the preoperative application of ICIs significantly improved the proportions of MPR and pCR. The pooled ES was 0.48 for MPR (95% CI 0.41–0.55; I²=87.96%) and 0.26 for pCR (95% CI 0.21–0.32; I²=86.49%) (**Figures 3A, B**). According to the Response Evaluation Criteria in Solid Tumors (RECIST 1.1) (50), the objective response was defined as the partial response (PR) and complete response (CR). After pooling the 35 studies (5, 6, 8–26, 28, 29, 31–49), the objective response rate (ORR) was found to be 0.57 (95% CI 0.47–0.68; I²=94.86%) (**Figure 3C**).

#### 3.2.2 Safety of treatment

TRAEs were graded using the Common Terminology Criteria for Adverse Events (CTCAE), with TRAEs graded ≥3 being considered serious adverse events (SAEs). Eighteen studies have assessed TRAEs (5, 6, 8–10, 13–16, 18–21, 23, 28, 30, 30, 34, 35, 40, 43, 47, 48), and their incidence ranges from 17–100%. The pooled ES was 0.70 (95% CI 0.56–0.81; I²=96.02%) (**Figure 3D**). Twenty-five studies have evaluated SAEs (5, 6, 8–16, 18–21, 25–28, 30, 31, 35–37, 39, 40, 42, 45–48), such as myelosuppression, gastrointestinal reactions, and immune-related target organ or tissue injury. The pooled ES was 0.17 (95% CI 0.10–0.25; I²=93.35%) (**Figure 3E**).

#### 3.2.3 Surgery

**Figure 4:**
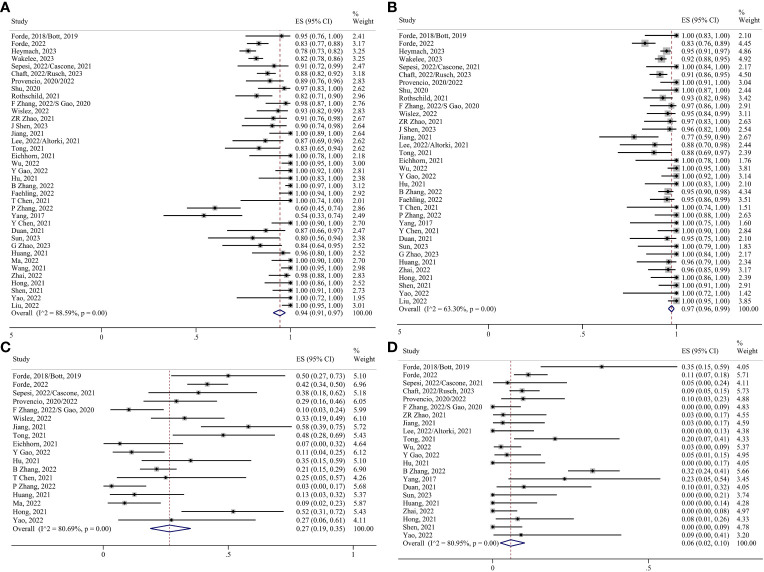
Forest plots. **(A)** Surgical resection. **(B)** R0 resection. **(C)** Surgical complications. **(D)** Conversion to thoracotomy.

Thirty-seven studies have evaluated surgical resection rates (5, 6, 8–38, 40–49), with the majority of patients undergoing surgery after neoadjuvant therapy (0.94, 95% CI 0.91–0.97; I²=88.59%) (**Figure 4A**). The pooled margin profile showed that most patients who underwent surgery could undergo R0 resection (0.97, 95% CI 0.96–0.99; I²=63.30%) (8–10, 13–18, 22–24, 29–38, 40, 41, 45–49) (**Figure 4B**). The main surgical complications included decreased hemoglobin levels, incisional infections, and prolonged air leaks. The pooled ES was 0.27 (95% CI 0.19–0.35; I²=80.69%) (5, 6, 8, 11, 12, 15, 16, 19–21, 24, 27, 28, 30–32, 34, 35, 42, 43, 46, 48) (**Figure 4C**). A few patients underwent conversions to thoracotomy due to the dense adhesion of blood vessels and lymph nodes after treatment (0.06, 95% CI 0.02–0.10; I²=80.95%) (5, 6, 8, 11–16, 19, 20, 22, 24–27, 29–32, 36, 38, 40, 42, 45–48) (**Figure 4D**).

### 3.3 Subgroup analysis

#### 3.3.1 Neoadjuvant immunotherapy alone and neoadjuvant chemoimmunotherapy

A subgroup analysis was performed according to the treatment regimen. In the ICIs-alone group, the heterogeneity of pCR, surgical resection, and R0 resection was significantly reduced, so the fixed effects model was used for the analysis, and the remaining indicators were analyzed using the random effects model. The pooled ES of MPR was 0.26 (95% CI 0.18-0.34; I²=54.73%); the pooled ES of pCR was 0.07 (95% CI 0.04-0.10; I²=28.90%) (**Supplementary Figure 1A**); the pooled ES of ORR was 0.13 (95% CI 0.07-0.22; I²=69.30%); the pooled ES of TRAEs was 0.43 (95% CI 0.28-0.58; I²=79.91%); the pooled ES of SAEs was 0.08 (95% CI 0.04-0.14; I²=53.85%); the pooled ES of surgical resection was 0.92 (95% CI 0.88-0.94; I²=17.75%) (**Supplementary Figure 1B**); the pooled ES of R0 resection 0.95 (95% CI 0.92-0.97; I²=16.12%) (**Supplementary Figure 1C**); the pooled ES of surgical complication was 0.27 (95% CI 0.14-0.41; I²=75.49%), the pooled ES of conversion to thoracotomy was 0.06 (95% CI 0.01-0.15; I²=79.00%).

**Figure 5:**
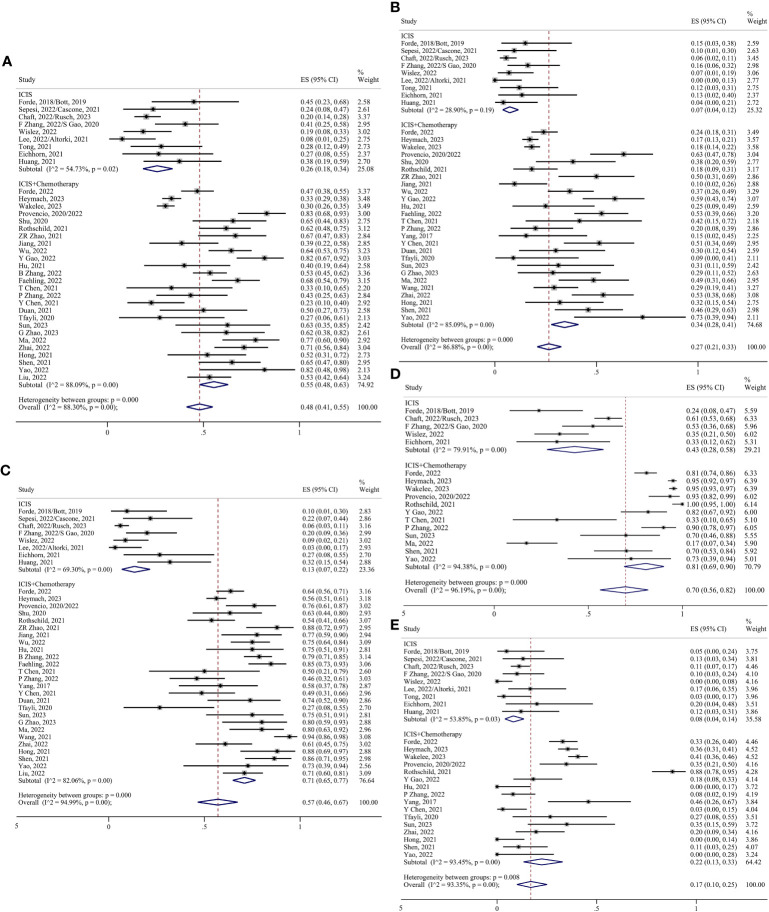
Forest plots of subgroups based on the treatment regimens. **(A)** MPR. **(B)** pCR. **(C)** Radiological response. **(D)** TRAEs. **(E)** SAEs.

**Figure 6:**
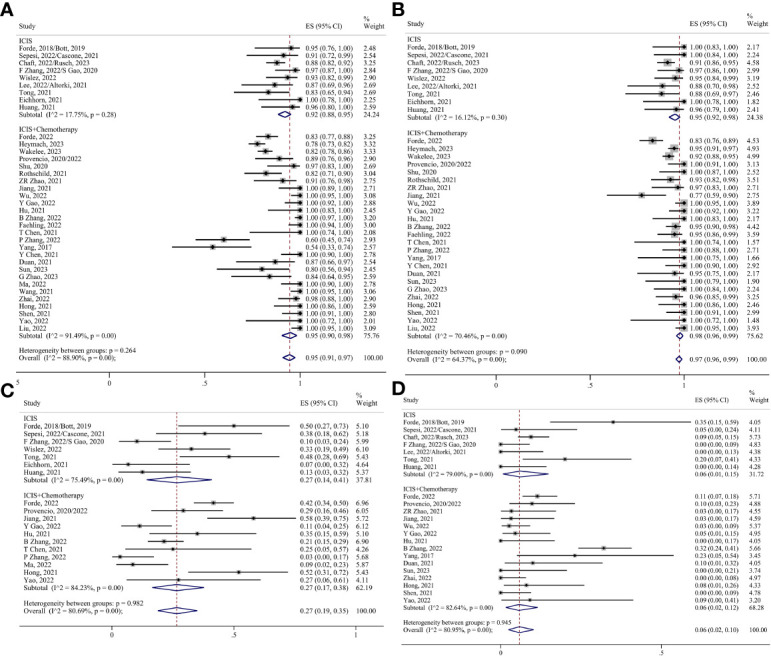
Forest plots of subgroups based on the treatment regimens. **(A)** Surgical resection. **(B)** R0 resection. **(C)** Surgical complications. **(D)** Conversion to thoracotomy.

In the chemoimmunotherapy group, each index was analyzed using the random effect model. The pooled ES of MPR was 0.55 (95% CI 0.48-0.63; I²=88.09%); the pooled ES of pCR was 0.34 (95% CI 0.28-0.41; I²=85.09%); the pooled ES of ORR was 0.71 (95% CI 0.65-0.77; I²=82.06%); the pooled ES of TRAEs was 0.81 (95% CI 0.69-0.90; I²=94.38%); the pooled ES of SAEs was 0.22 (95% CI 0.13-0.33; I²=93.45%); the pooled ES of surgical resection was 0.95 (95% CI 0.90-0.98; I²=91.49%); the pooled ES of R0 resection 0.98 (95% CI 0.96-0.99; I²=70.46%); the pooled ES of surgical complication was 0.27 (95% CI 0.17-0.38; I²=84.23%); the pooled ES of conversion to thoracotomy was 0.06 (95% CI 0.02-0.12; I²=82.64%) (**Figures 5**, **6**).

#### 3.3.2 Neoadjuvant treatment cycle

The studies were divided into two-cycle, three-cycle, and four-cycle groups according to the number of neoadjuvant therapy cycles. Thirty-seven studies reported on treatment cycles, including 12 studies in the two-cycle group, 7 studies in the three-cycle group, 4 studies in the four-cycle group, and the rest of the studies could not be subgrouped. All studies in the four-cycle group were chemoimmunotherapy. Analyses will be stratified according to the neoadjuvant regimen.

**Figure 7:**
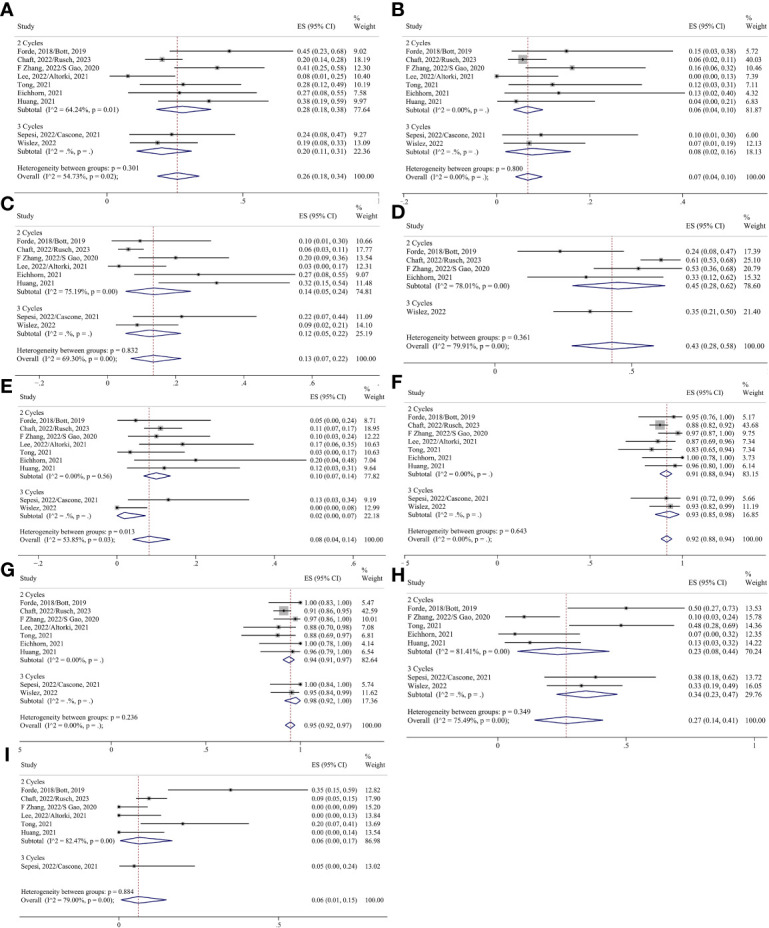
Forest plots of subgroups based on immunotherapy-alone treatment cycles. **(A)** MPR. **(B)** pCR. **(C)** Radiological response. **(D)** TRAEs. **(E)** SAEs. **(F)** Surgical resection. **(G)** R0 resection. **(H)** Surgical complications. **(I)** Conversion to thoracotomy.

For neoadjuvant immunotherapy alone, pCR, SAEs, R0 resection, and surgery resection were analyzed by the fixed effect model, and other indicators were analyzed by the random effect model. The ES for MPR in the two-cycle and three-cycle groups were 0.28 (95% CI 0.18-0.38) and 0.20 (95% CI 0.11-0.31); for pCR, they were 0.06 (95% CI 0.04-0.10) and 0.08 (95% CI 0.02-0.16); for ORR, they were 0.14 (95% CI 0.05-0.24) and 0.12 (95% CI 0.05-0.22); for TRAEs, they were 0.45 (95% CI 0.28-0.62) and 0.35 (95% CI 0.21-0.50); for SAEs, they were 0.10 (95% CI 0.07-0.14) and 0.02 (95% CI 0.00-0.07); for surgery resection, they were 0.91 (95% CI 0.88-0.94) and 0.93 (95% CI 0.85-0.98); for R0 resection, they were 0.94 (95% CI 0.91-0.97) and 0.98 (95% CI 0.92-1.00); for surgical complication, they were 0.23 (95% CI 0.08-0.44) and 0.34 (95% CI 0.23-0.47); and for conversion to thoracotomy, they were 0.06 (95% CI 0.00-0.17) and 0.05 (95% CI 0.00-0.24), respectively (**Figure 7**).

**Figure 8:**
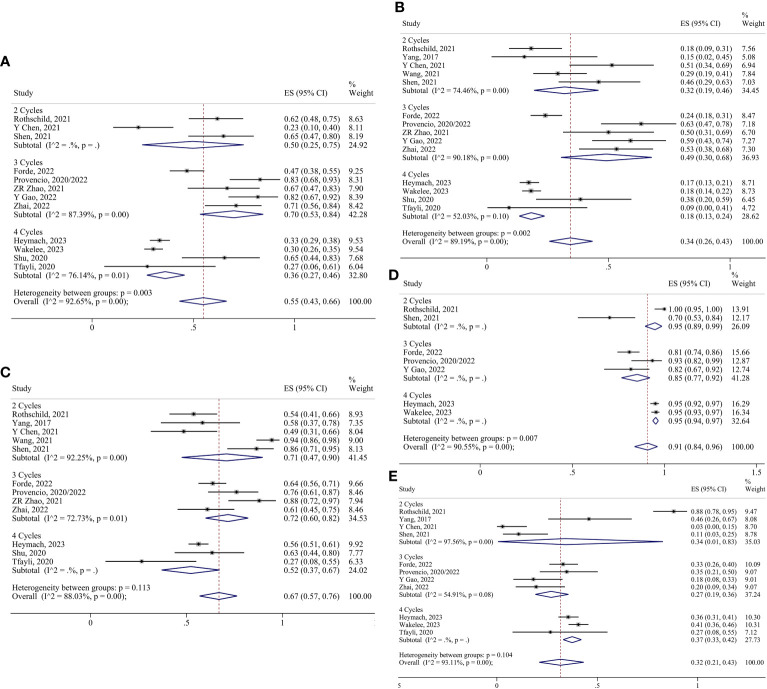
Forest plots of subgroups based on chemoimmunotherapy treatment cycles. **(A)** MPR. **(B)** pCR. **(C)** Radiological response. **(D)** TRAEs. **(E)** SAEs.

**Figure 9:**
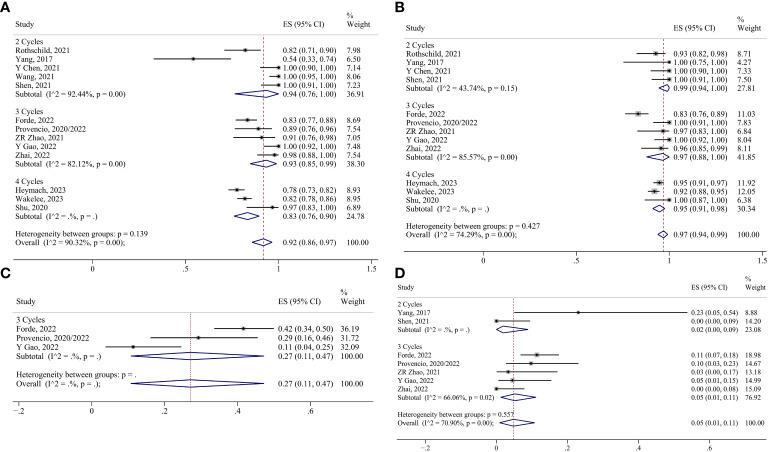
Forest plots of subgroups based on chemoimmunotherapy treatment cycles. **(A)** Surgical resection. **(B)** R0 resection. **(C)** Surgical complications. **(D)** Conversion to thoracotomy.

For neoadjuvant chemoimmunotherapy, the R0 resection rate in the two-cycle group was analyzed using the fixed effects model. The random-effects model was adopted for the rest of the indicators. No reports of conversion to thoracotomy were found in the four-cycle group, and no reports of surgical complication rates were found in the two-cycle group or the four-cycle group. Consequently, the ES for conversion to thoracotomy in the two-cycle and three-cycle group were 0.02 (95% CI 0.00-0.09) and 0.05 (95% CI 0.01-0.11), respectively; and the ES for surgical complication in the three-cycle group was 0.27 (95% CI 0.11-0.47). The ES for MPR in the two-cycle, three-cycle and four-cycle groups were 0.50 (95% CI 0.25-0.75), 0.70 (95% CI 0.53-0.84) and 0.36 (95% CI 0.27-0.46); for pCR, they were 0.32 (95% CI 0.19-0.46), 0.49 (95% CI 0.30-0.68) and 0.18 (95% CI 0.13-0.24); for ORR, they were 0.71 (95% CI 0.47-0.90), 0.72 (95% CI 0.60-0.82) and 0.52 (95% CI 0.37-0.67);for TRAEs, they were 0.95 (95% CI 0.89-0.99), 0.85 (95% CI 0.77-0.92) and 0.95 (95% CI 0.94-0.97); for SAEs, they were 0.34 (95% CI 0.01-0.83), 0.27 (95% CI 0.19-0.36) and 0.37 (95% CI 0.33-0.42); for surgery resection, they were 0.94 (95% CI 0.76-1.00), 0.93 (95% CI 0.85-0.99) and 0.83 (95% CI 0.76-0.90); for R0 resection, they were 0.99 (95% CI 0.95-1.00) (**Supplementary Figure 1D**), 0.97 (95% CI 0.88-1.00) and 0.95 (95% CI 0.91-0.98), respectively (**Figures 8**, **9**).

### 3.4 Sensitivity analysis and publication bias

Upon examining the effects of different studies on heterogeneity within the subgroups, the heterogeneity of the chemoimmunotherapy group converted to thoracotomy after deletion of Zhang et al., 2022 (28) was significantly reduced (I²=52.03%, P=0.01), with an effect size of 0.04 (95% CI 0.01-0.07). For the neoadjuvant cycle subgroup analysis, the study by Rothschild et al., 2021 (15) was excluded from the chemoimmunotherapy group. The ES of the TRAEs in the two-cycle group changed to 0.70 (95% CI 0.53–0.84), and that of the SAEs changed to 0.16 (95% CI 0.01–0.44). Egger’s tests (**Supplementary Table 2**) and funnel plots (**Supplementary Figure 2, 3**) were performed separately within the different subgroups, and there was no marked publication bias.

## 4 Discussion

This study demonstrated the feasibility and safety of the preoperative application of ICIs. Subgroup and meta-regression analyses (**Supplementary Table 3**) showed that chemoimmunotherapy increased tumor MPR and pCR rates by 29% and 27% compared with ICIs alone (*P*= 0.001; *P*=0.002), respectively; while the ORR increased significantly (*P* < 0.001). However, the incidence of TRAEs and SAEs increased significantly (*P* = 0.009; *P*=0.034). All phase III large-sample clinical trials on chemoimmunotherapy reported on SAEs, which were dominated by Neutrophil count decreased, neutropenia, anemia, leukopenia, and Platelet count decreased (8–10). None of them found significant differences in TRAEs and SAEs between chemoimmunotherapy and chemotherapy. This suggests that the increased incidence of TRAEs and SAEs with chemoimmunotherapy compared to immunotherapy alone may be related to chemotherapy. AEGEAN (9) and KEYNOTE-671 (10) reported 7 and 4 deaths during the neoadjuvant therapy phase, respectively, with the main causes of death being immune-mediated lung disease, interstitial lung disease and pneumonia. Despite the low mortality associated with chemoimmunotherapy, physicians still need to be vigilant for the occurrence of immune-related diseases, especially immune-mediated lung disease. In terms of surgery, combination chemotherapy did not significantly affect surgical resection, R0 resection, conversion to thoracotomy, or surgical complications.

Among the included studies, CheckMate816 and NADIM II reported data related to circulating tumor DNA (ctDNA). There was a ctDNA clearance rate of 56% in the neoadjuvant chemotherapy group (8). Both reported that higher clearance was associated with a higher rate of pCR and longer event-free survival (EFS) (8, 16). Although follow-up data, such as five-year overall survival (OS), have not been reported, higher ctDNA clearance rates are beneficial for predicting the long-term risks of neoadjuvant immunotherapy (51, 52).

In terms of treatment cycles, for chemoimmunotherapy, MPR and pCR were improved by 20% and 17% in the three-cycle group compared with the two-cycle group, respectively, but there was no increase in the MPR or pCR for the four-cycle group. Similarly, the neoSCORE study reported a 14.5% increase in the MPR and a 4.9% increase in the pCR in a three-cycle group compared with the two-cycle group with preoperative sintilimab combined with platinum-based dual chemotherapy regimens (53). After sensitivity analysis, the incidence of TRAEs and SAEs in the chemoimmunotherapy group increased progressively with the number of treatment cycles, but none of them was statistically significant. Therefore, three cycles of neoadjuvant chemoimmunotherapy have an optimal efficacy and safety profile compared to two and four cycles. In the ICIs-alone group, the increase of treatment cycles had little effect on the rate of MPR and pCR, the incidence of TRAEs and SAEs. There was no significant negative effect of the increase of neoadjuvant cycles on the rate of surgical resection, the incidence of surgical complications, rate of R0 resection, or rate of conversion to thoracotomy in both treatment regimens.

The preoperative application of ICIs is not limited to combination chemotherapy. Compared with neoadjuvant chemotherapy, neoadjuvant radiation therapy combined with chemotherapy for lung cancer does not produce long-term benefits in terms of EFS and OS and has more significant side effects (54, 55). However, Lee et al., 2022 (25) and Altorki et al., 2021 (26) noted that the MPR rate in groups treated with durvalumab combined with SBRT was 53.3%, which was significantly higher than that in groups treated with durvalumab alone. The efficacy of nivolumab in combination with ipilimumab has been confirmed in NEOSTAR; however, there are few relevant studies on this treatment, and further analyses in large-sample studies are required.

This meta-analysis clarified the safety and feasibility of different neoadjuvant regimens and cycles at the present stage and provides a reference for the selection of regimens and cycles. However, there were several limitations. First, only three phase III large-sample clinical trials and a large number of phase II single-arm studies were included. The conclusions of the study are therefore unrepresentative and inaccurate. Second, the heterogeneity of the outcomes was strong after pooling. The heterogeneity of some of the results decreased insignificantly after the subgroup analysis, and there was a lack of long-term follow-up data. Third, studies at this stage have mainly focused on ICIs alone and chemoimmunotherapy. We look forward to clinical studies on ICIs combined with radiotherapy, targeted therapy, or dual immunotherapy to determine the optimal neoadjuvant treatment strategy for lung cancer.

## Data availability statement

The original contributions presented in the study are included in the article/**Supplementary Material**. Further inquiries can be directed to the corresponding author.

## Author contributions

HW: Conceptualization, Data curation, Formal analysis, Investigation, Methodology, Software, Visualization, Writing – original draft, Writing – review & editing. SL: Data curation, Formal analysis, Investigation, Writing – review & editing. YY: Data curation, Validation, Writing – review & editing. YH: Conceptualization, Data curation, Funding acquisition, Investigation, Methodology, Resources, Supervision, Writing – review & editing.
