# Supplementary material for: Efficacy and safety of neoadjuvant immunotherapy protocols and cycles for non-small cell lung cancer: a systematic review and meta-analysis
Source: Front Oncol. 2024 Jan 16;14:1276549. doi: 10.3389/fonc.2024.1276549 (PMC10824986; doi:10.3389/fonc.2024.1276549)
Supplement: Supplementary file 1 [file DataSheet_1.doc]

Supplementary Material

# Supplementary Data

**Supplementary Table 1.** Literature evaluation of MINORS.

**Supplementary Table 2.** *P* value of Subgroup Egger's test.

**Supplementary Table 3.** *P* value of Subgroup Meta-Regression.

**Supplementary Figure 1.** Fixed effect forest plots. **(A)** pCR of ICIs group. **(B)** Surgical resection of ICIs group. **(C)** R0 resection of ICIs group **(D)** R0 resection in two cycles of chemoimmunotherapy group.

**Supplementary Figure 2.** Funnel plot of ICIs group. **(A)** MPR. **(B)** pCR. **(C)** Radiological response. **(D)** TRAEs. **(E)** SAEs. **(F)** Surgical resection. **(G)** R0 resection. **(H)** Surgical complications. **(I)** Conversion to thoracotomy.

**Supplementary Figure 3.** Funnel plot of chemoimmunotherapy group. **(A)** MPR. **(B)** pCR. **(C)** Radiological response. **(D)** TRAEs. **(E)** SAEs. **(F)** Surgical resection. **(G)** R0 resection. **(H)** Surgical complications. **(I)** Conversion to thoracotomy.

**Search strategy**

# Supplementary Figures and Tables

**Supplementary Table 1.** Literature evaluation of MINORS.

| Study | a | b | c | d | e | f | g | h | i | j | k | l | Total | |
| --- | --- | --- | --- | --- | --- | --- | --- | --- | --- | --- | --- | --- | --- | --- |
| Forde et al. | 2 | 2 | 2 | 2 | 0 | 1 | 2 | 0 | - | - | - | - | 11 | |
| Bott et al. | 2 | 2 | 2 | 2 | 0 | 1 | 2 | 0 | - | - | - | - | 11 | |
| Chaft et al. | 2 | 2 | 2 | 2 | 0 | 1 | 2 | 1 | - | - | - | - | 12 | |
| Rusch et al. | 2 | 2 | 2 | 2 | 0 | 1 | 2 | 1 |  |  |  |  | 12 | |
| Provencio et al. | 2 | 2 | 2 | 2 | 1 | 1 | 2 | 1 | - | - | - | - | 13 | |
| Provencio et al. | 2 | 2 | 2 | 2 | 1 | 1 | 2 | 1 | - | - | - | - | 13 | |
| Shu et al. | 2 | 2 | 2 | 2 | 0 | 1 | 2 | 1 | - | - | - | - | 12 | |
| Rothschild et al. | 2 | 2 | 2 | 2 | 0 | 1 | 2 | 1 | - | - | - | - | 12 | |
| F Zhang et al. | 2 | 2 | 2 | 2 | 0 | 1 | 2 | 0 | - | - | - | - | 11 | |
| S Gao et al. | 2 | 2 | 2 | 2 | 0 | 1 | 2 | 0 | - | - | - | - | 11 | |
| Wislez et al. | 2 | 2 | 2 | 2 | 0 | 1 | 2 | 1 | - | - | - | - | 12 | |
| ZR Zhao et al. | 2 | 2 | 2 | 2 | 0 | 1 | 2 | 1 | - | - | - | - | 12 | |
| J Shen et al. | 2 | 2 | 2 | 2 | 0 | 1 | 2 | 0 | 2 | 2 | 2 | 2 | 19 | |
| Jiang et al. | 2 | 2 | 2 | 2 | 0 | 0 | 0 | 0 | - | - | - | - | 8 | |
| Tong et al. | 2 | 2 | 2 | 2 | 0 | 1 | 2 | 0 | - | - | - | - | 11 | |
| Eichhorn et al. | 2 | 2 | 2 | 2 | 0 | 0 | 0 | 0 | - | - | - | - | 8 | |
| Wu et al. | 2 | 2 | 2 | 2 | 0 | 1 | 2 | 0 | - | - | - | - | 11 | |
| Y Gao et al. | 2 | 2 | 2 | 2 | 0 | 0 | 0 | 0 | - | - | - | - | 8 | |
| Hu et al. | 2 | 2 | 2 | 2 | 0 | 0 | 0 | 0 | - | - | - | - | 8 | |
| B Zhang et al. | 2 | 2 | 2 | 2 | 0 | 1 | 2 | 0 | - | - | - | - | 11 | |
| Faehling et al. | 2 | 2 | 2 | 2 | 0 | 1 | 2 | 0 | - | - | - | - | 11 | |
| T Chen et al. | 2 | 2 | 2 | 2 | 0 | 1 | 2 | 0 | - | - | - | - | 11 | |
| P Zhang et al. | 2 | 2 | 2 | 2 | 0 | 1 | 2 | 0 | - | - | - | - | 11 | |
| Yang et al. | 2 | 2 | 2 | 2 | 0 | 1 | 2 | 0 | - | - | - | - | 11 | |
| Y Chen et al. | 2 | 2 | 2 | 2 | 0 | 1 | 2 | 0 | - | - | - | - | 11 | |
| Duan et al. | 2 | 2 | 2 | 2 | 0 | 1 | 2 | 0 | - | - | - | - | 11 | |
| Tfayli et al. | 2 | 2 | 2 | 2 | 0 | 0 | 0 | 1 |  |  |  |  | 9 | |
| Sun et al. | 2 | 2 | 2 | 2 | 0 | 1 | 2 | 0 | - | - | - | - | 11 | |
| G Zhao et al. | 2 | 2 | 2 | 2 | 0 | 0 | 0 | 0 |  |  |  |  | 8 | |
| Huang et al. | 2 | 2 | 2 | 2 | 1 | 0 | 0 | 0 | 2 | 2 | 2 | 2 | 17 | |
| Ma et al. | 2 | 2 | 2 | 2 | 0 | 0 | 0 | 0 | - | - | - | - | 8 | |
| Wang et al. | 2 | 2 | 2 | 2 | 0 | 0 | 0 | 0 | - | - | - | - | 8 | |
| Zhai et al. | 2 | 2 | 2 | 2 | 0 | 1 | 2 | 0 | - | - | - | - | 11 | |
| Hong et al. | 2 | 2 | 2 | 2 | 0 | 0 | 0 | 0 | - | - | - | - | 8 | |
| Shen et al. | 2 | 2 | 2 | 2 | 0 | 1 | 2 | 0 | - | - | - | - | 11 | |
| Yao et al. | 2 | 2 | 2 | 2 | 0 | 1 | 2 | 0 | - | - | - | - | 11 | |
| Liu et al. | 2 | 2 | 2 | 2 | 0 | 1 | 2 | 0 | - | - | - | - | 11 | |
| a, A clearly stated aim; b, Inclusion of consecutive patients; c, Prospective collection of data; d, Endpoint appropriate to the study aim; e, Unbiased assessment of endpoints; f, Follow-up period appropriate to the major endpoint; g, Loss to follow up not exceeding 5%; h, Prospective calculation of the sample size; i, Selection of appropriate control group; j, Contemporary groups; k, Baseline equivalence of groups; l, Statistical analyses adapted to study design. | | | | | | | | | | | | | |  |

**Supplementary Table 2.** *P* value of Subgroup Egger's test.

| Subgroups | MPR | pCR | PR+CR | TRAEs | SAE | Surgical resection | R0 resection | Surgical  complications | Conversion to thoracotomy |
| --- | --- | --- | --- | --- | --- | --- | --- | --- | --- |
| ICIs | 0.279 | 0.270 | 0.068 | 0.050 | 0.988 | 0.141 | 0.122 | 0.799 | 0.849 |
| ICIs + Chemo | 0.904 | 0.475 | 0.225 | 0.065 | 0.112 | 0.163 | 0.079 | 0.765 | 0.086 |

**Supplementary Table 3.** *P* value of Subgroup Meta-Regression.

| Subgroups | MPR | pCR | PR+CR | TRAEs | SAE | Surgical resection | R0 resection | Surgical  complications | Conversion to thoracotomy |
| --- | --- | --- | --- | --- | --- | --- | --- | --- | --- |
| Protocols | 0.001 | 0.002 | <0.001 | 0.009 | 0.034 | 0.703 | 0.875 | 0.931 | 0.783 |
| Cycles |  |  |  |  |  |  |  |  |  |
| ICIs | 0.696 | 0.993 | 0.877 | 0.436 | 0.638 | 0.845 | 0.775 | 0.543 | NA |
| ICIs + Chemo | 0.255 | 0.215 | 0.172 | 0.253 | 0.848 | 0.148 | 0.875 | NA | 0.918 |

NA: not available


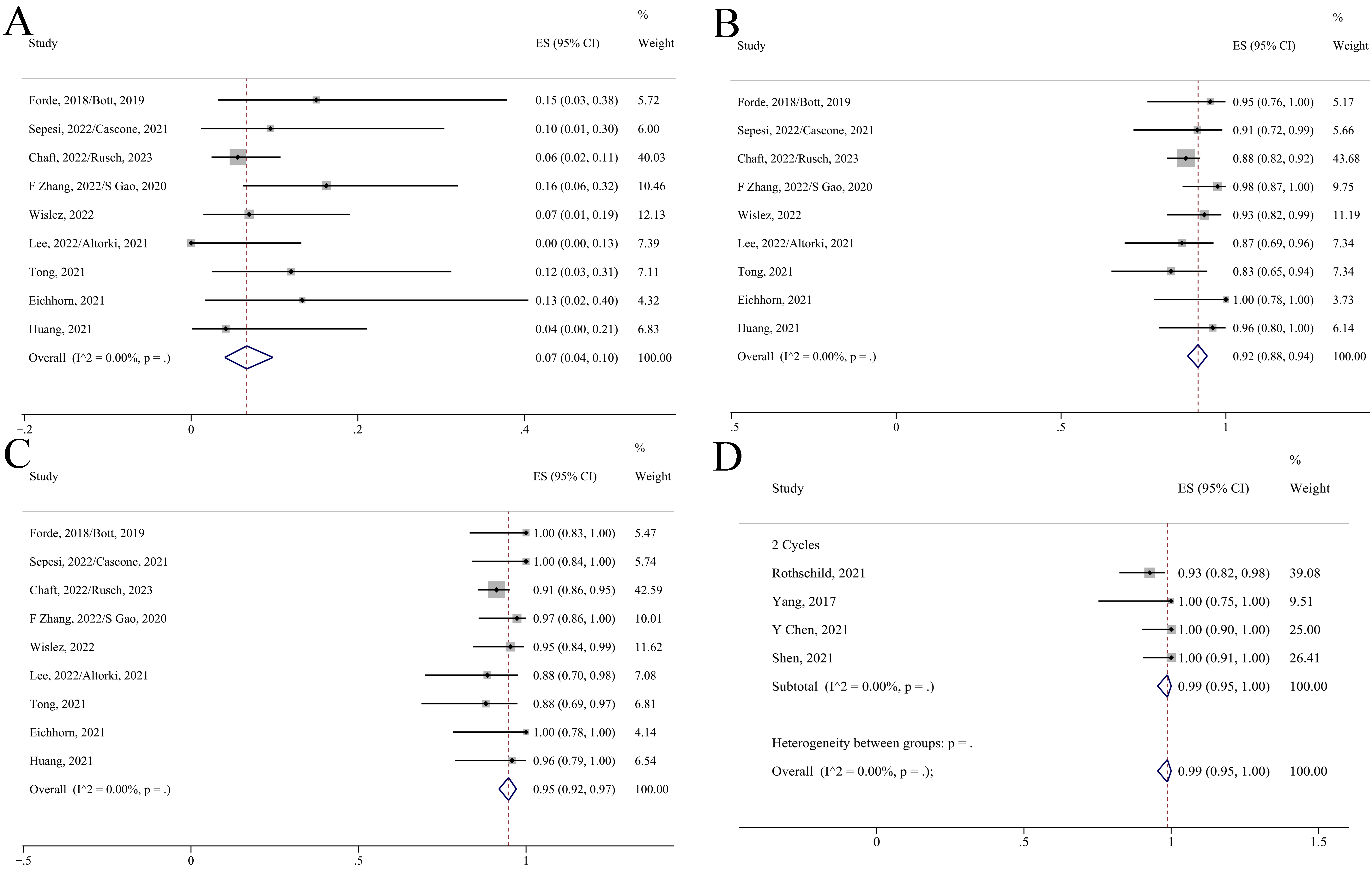
**Supplementary Figure 1.** Fixed effect forest plots. **(A)** pCR of ICIs group. **(B)** Surgical resection of ICIs group. **(C)** R0 resection of ICIs group **(D)** R0 resection in two cycles of chemoimmunotherapy group.


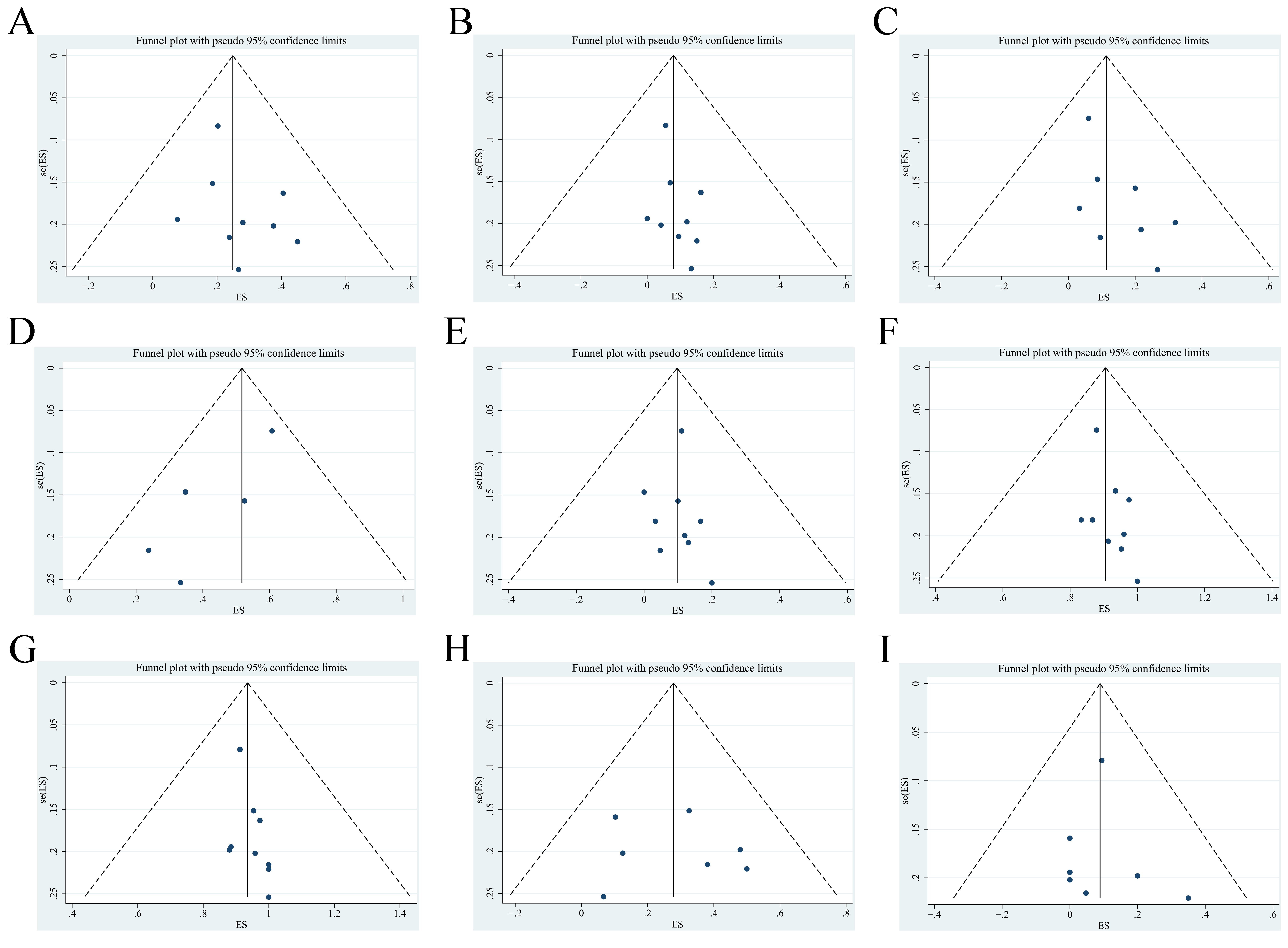


**Supplementary Figure 2.** Funnel plot of ICIs group. **(A)** MPR. **(B)** pCR. **(C)** Radiological response. **(D)** TRAEs. **(E)** SAEs. **(F)** Surgical resection. **(G)** R0 resection. **(H)** Surgical complications. **(I)** Conversion to thoracotomy.


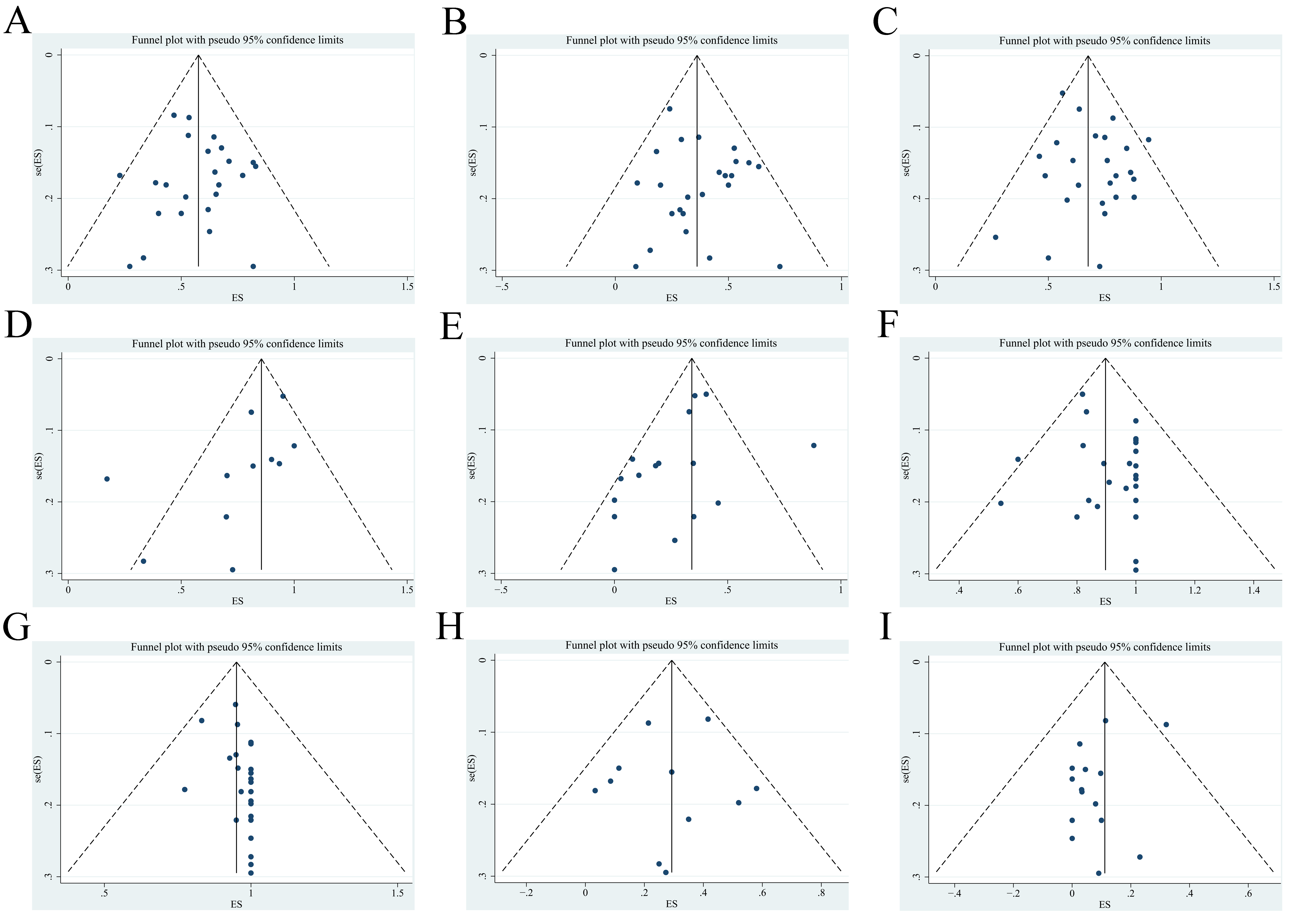


**Supplementary Figure 3.** Funnel plot of chemoimmunotherapy group. **(A)** MPR. **(B)** pCR. **(C)** Radiological response. **(D)** TRAEs. **(E)** SAEs. **(F)** Surgical resection. **(G)** R0 resection. **(H)** Surgical complications. **(I)** Conversion to thoracotomy.

CI, confidence interval; CR, complete response; ES, effect size; I2, inconsistency index; ICIs, immune checkpoint inhibitors; MPR, major pathologic response; pCR, complete pathologic response; PR, partial response; SAEs, serious adverse events; TRAEs, treatment-related adverse events.

# Search strategy

**PubMed**

((("Carcinoma, Non-Small-Cell Lung"[Mesh]) OR (((((((((((((Carcinoma, Non Small Cell Lung[Title/Abstract]) OR (Carcinomas, Non-Small-Cell Lung[Title/Abstract])) OR (Lung Carcinoma, Non-Small-Cell[Title/Abstract])) OR (Lung Carcinomas, Non-Small-Cell[Title/Abstract])) OR (Non-Small-Cell Lung Carcinomas[Title/Abstract])) OR (Non-Small-Cell Lung Carcinoma[Title/Abstract])) OR (Non Small Cell Lung Carcinoma[Title/Abstract])) OR (Carcinoma, Non-Small Cell Lung[Title/Abstract])) OR (Non-Small Cell Lung Carcinoma[Title/Abstract])) OR (Non-Small Cell Lung Cancer[Title/Abstract])) OR (Nonsmall Cell Lung Cancer[Title/Abstract])) OR (NSCLC[Title/Abstract])) OR (nsclc[Title/Abstract]))) AND ((Immunotherapies[Title/Abstract]) OR ("Immunotherapy"[Mesh]))) AND (("Neoadjuvant Therapy"[Mesh]) OR ((((((((((((((((((((((((((((((((((((((((((((((((Neoadjuvant Therapies[Title/Abstract]) OR (Therapy, Neoadjuvant[Title/Abstract])) OR (Neoadjuvant Treatment[Title/Abstract])) OR (Neoadjuvant Treatments[Title/Abstract])) OR (Treatment, Neoadjuvant[Title/Abstract])) OR (Neoadjuvant Radiotherapy[Title/Abstract])) OR (Neoadjuvant Radiotherapies[Title/Abstract])) OR (Radiotherapy, Neoadjuvant[Title/Abstract])) OR (Neoadjuvant Radiation Treatment[Title/Abstract])) OR (Neoadjuvant Radiation Treatments[Title/Abstract])) OR (Radiation Treatment, Neoadjuvant[Title/Abstract])) OR (Treatment, Neoadjuvant Radiation[Title/Abstract])) OR (Neoadjuvant Radiation Therapy[Title/Abstract])) OR (Neoadjuvant Radiation Therapies[Title/Abstract])) OR (Radiation Therapy, Neoadjuvant[Title/Abstract])) OR (Therapy, Neoadjuvant Radiation[Title/Abstract])) OR (Neoadjuvant Radiation[Title/Abstract])) OR (Neoadjuvant Radiations[Title/Abstract])) OR (Radiation, Neoadjuvant[Title/Abstract])) OR (Neoadjuvant Systemic Therapy[Title/Abstract])) OR (Neoadjuvant Systemic Therapies[Title/Abstract])) OR (Systemic Therapy, Neoadjuvant[Title/Abstract])) OR (Therapy, Neoadjuvant Systemic[Title/Abstract])) OR (Neoadjuvant Systemic Treatment[Title/Abstract])) OR (Neoadjuvant Systemic Treatments[Title/Abstract])) OR (Systemic Treatment, Neoadjuvant[Title/Abstract])) OR (Treatment, Neoadjuvant Systemic[Title/Abstract])) OR (Neoadjuvant Chemotherapy[Title/Abstract])) OR (Chemotherapy, Neoadjuvant[Title/Abstract])) OR (Neoadjuvant Chemotherapies[Title/Abstract])) OR (Neoadjuvant Chemotherapy Treatment[Title/Abstract])) OR (Chemotherapy Treatment, Neoadjuvant[Title/Abstract])) OR (Neoadjuvant Chemotherapy Treatments[Title/Abstract])) OR (Treatment, Neoadjuvant Chemotherapy[Title/Abstract])) OR (Neoadjuvant Chemoradiotherapy[Title/Abstract])) OR (Chemoradiotherapy, Neoadjuvant[Title/Abstract])) OR (Neoadjuvant Chemoradiotherapies[Title/Abstract])) OR (Neoadjuvant Chemoradiation Therapy[Title/Abstract])) OR (Chemoradiation Therapy, Neoadjuvant[Title/Abstract])) OR (Neoadjuvant Chemoradiation Therapies[Title/Abstract])) OR (Therapy, Neoadjuvant Chemoradiation[Title/Abstract])) OR (Neoadjuvant Chemoradiation Treatment[Title/Abstract])) OR (Chemoradiation Treatment, Neoadjuvant[Title/Abstract])) OR (Neoadjuvant Chemoradiation Treatments[Title/Abstract])) OR (Treatment, Neoadjuvant Chemoradiation[Title/Abstract])) OR (Neoadjuvant Chemoradiation[Title/Abstract])) OR (Chemoradiation, Neoadjuvant[Title/Abstract])) OR (Neoadjuvant Chemoradiations[Title/Abstract])))

**Embase**

#10 #3 AND #6 AND #9

#9 #7 OR #8

#8 'immunotherapies':ab,ti

#7 'immunotherapy'/exp

#6 #4 OR #5

#5 ‘neoadjuvant therapies’:ab,ti OR ‘therapy, neoadjuvant’:ab,ti OR ‘neoadjuvant treatment’:ab,ti OR ‘neoadjuvant treatments’:ab,ti OR ‘treatment, neoadjuvant’:ab,ti OR ‘neoadjuvant radiotherapy’:ab,ti OR ‘radiotherapy, neoadjuvant’:ab,ti OR ‘neoadjuvant radiation treatments’:ab,ti OR ‘neoadjuvant radiotherapies’:ab,ti OR ‘neoadjuvant radiation treatment’:ab,ti OR ‘radiation treatment, neoadjuvant’:ab,ti OR ‘treatment, neoadjuvant radiation’:ab,ti OR ‘neoadjuvant radiation therapy’:ab,ti OR ‘radiation therapy, neoadjuvant’:ab,ti OR ‘neoadjuvant radiation therapies’:ab,ti OR ‘therapy, neoadjuvant radiation’:ab,ti OR ‘neoadjuvant radiation’:ab,ti OR ‘neoadjuvant radiations’:ab,ti OR ‘radiation, neoadjuvant’:ab,ti OR ‘neoadjuvant systemic therapy’:ab,ti OR ‘systemic therapy, neoadjuvant’:ab,ti OR ‘therapy, neoadjuvant systemic’:ab,ti OR ‘neoadjuvant systemic therapies’:ab,ti OR ‘neoadjuvant systemic treatments’:ab,ti OR ‘neoadjuvant systemic treatment’:ab,ti OR ‘systemic treatment, neoadjuvant’:ab,ti OR ‘treatment, neoadjuvant systemic’:ab,ti OR ‘neoadjuvant chemotherapy’:ab,ti OR ‘chemotherapy, neoadjuvant’:ab,ti OR ‘neoadjuvant chemotherapies’:ab,ti OR ‘neoadjuvant chemotherapy treatment’:ab,ti OR ‘chemotherapy treatment, neoadjuvant’:ab,ti OR ‘neoadjuvant chemotherapy treatments’:ab,ti OR ‘treatment, neoadjuvant chemotherapy’:ab,ti OR ‘neoadjuvant chemoradiotherapy’:ab,ti OR ‘chemoradiotherapy, neoadjuvant’:ab,ti OR ‘neoadjuvant chemoradiotherapies’:ab,ti OR ‘neoadjuvant chemoradiation therapy’:ab,ti OR ‘chemoradiation therapy, neoadjuvant’:ab,ti OR ‘neoadjuvant chemoradiation therapies’:ab,ti OR ‘neoadjuvant chemoradiation treatment’:ab,ti OR ‘therapy, neoadjuvant chemoradiation’:ab,ti OR ‘neoadjuvant chemoradiation treatments’:ab,ti OR ‘chemoradiation treatment, neoadjuvant’:ab,ti OR ‘treatment, neoadjuvant chemoradiation’:ab,ti OR ‘neoadjuvant chemoradiation’:ab,ti OR ‘chemoradiation, neoadjuvant’:ab,ti OR ‘neoadjuvant chemoradiations’:ab,ti

#4 'neoadjuvant therapy'/exp

#3 #1 OR #2

#2 'carcinoma, non small cell lung':ab,ti OR 'carcinomas, non-small-cell lung':ab,ti OR 'lung carcinoma, non-small-cell':ab,ti OR 'lung carcinomas, non-small-cell':ab,ti OR 'non-small-cell lung carcinomas':ab,ti OR 'non-small-cell lung carcinoma':ab,ti OR 'carcinoma, non-small cell lung':ab,ti OR 'non-small cell lung cancer':ab,ti OR 'non small cell lung carcinoma':ab,ti OR 'non-small cell lung carcinoma':ab,ti OR 'nonsmall cell lung cancer':ab,ti OR 'nsclc':ab,ti

#1 'non small cell lung cancer'/exp

**Cochrane Library**

#1 Carcinoma, Non-Small-Cell Lung

#2 (Carcinoma, Non Small Cell Lung):ti,ab,kw OR (Carcinomas, Non-Small-Cell Lung):ti,ab,kw OR (Lung Carcinoma, Non-Small-Cell):ti,ab,kw OR (Lung Carcinomas, Non-Small-Cell):ti,ab,kw OR (Non-Small-Cell Lung Carcinomas):ti,ab,kw OR (Non-Small-Cell Lung Carcinoma):ti,ab,kw OR (Non Small Cell Lung Carcinoma):ti,ab,kw OR (Carcinoma, Non-Small Cell Lung):ti,ab,kw OR (Non-Small Cell Lung Carcinoma):ti,ab,kw OR (Non-Small Cell Lung Cancer):ti,ab,kw OR (Nonsmall Cell Lung Cancer):ti,ab,kw OR (NSCLC):ti,ab,kw OR (nsclc):ti,ab,kw

#3 #1 or #2

#4 Neoadjuvant Therapy

#5 (Neoadjuvant Therapies ):ti,ab,kw OR (Therapy, Neoadjuvant):ti,ab,kw OR (Neoadjuvant Treatment):ti,ab,kw OR (Neoadjuvant Treatments):ti,ab,kw OR (Treatment, Neoadjuvant):ti,ab,kw OR (Neoadjuvant Radiotherapy):ti,ab,kw OR (Neoadjuvant Radiotherapies):ti,ab,kw OR (Radiotherapy, Neoadjuvant):ti,ab,kw OR (Neoadjuvant Radiation Treatment):ti,ab,kw OR (Neoadjuvant Radiation Treatments):ti,ab,kw OR (Radiation Treatment, Neoadjuvant):ti,ab,kw OR (Treatment, Neoadjuvant Radiation):ti,ab,kw OR (Neoadjuvant Radiation Therapy):ti,ab,kw OR (Neoadjuvant Radiation Therapies):ti,ab,kw OR (Radiation Therapy, Neoadjuvant):ti,ab,kw OR (Therapy, Neoadjuvant Radiation):ti,ab,kw OR (Neoadjuvant Radiation):ti,ab,kw OR (Neoadjuvant Radiations):ti,ab,kw OR (Radiation, Neoadjuvant):ti,ab,kw OR (Neoadjuvant Systemic Therapy):ti,ab,kw OR (Neoadjuvant Systemic Therapies):ti,ab,kw OR (Systemic Therapy, Neoadjuvant):ti,ab,kw OR (Therapy, Neoadjuvant Systemic):ti,ab,kw OR (Neoadjuvant Systemic Treatment):ti,ab,kw OR (Neoadjuvant Systemic Treatments):ti,ab,kw OR (Systemic Treatment, Neoadjuvant):ti,ab,kw OR (Treatment, Neoadjuvant Systemic):ti,ab,kw OR (Neoadjuvant Chemotherapy):ti,ab,kw OR (Chemotherapy, Neoadjuvant):ti,ab,kw OR (Neoadjuvant Chemotherapies):ti,ab,kw OR (Neoadjuvant Chemotherapy Treatment):ti,ab,kw OR (Chemotherapy Treatment, Neoadjuvant):ti,ab,kw OR (Neoadjuvant Chemotherapy Treatments):ti,ab,kw OR (Treatment, Neoadjuvant Chemotherapy):ti,ab,kw OR (Neoadjuvant Chemoradiotherapy):ti,ab,kw OR (Chemoradiotherapy, Neoadjuvant):ti,ab,kw OR (Neoadjuvant Chemoradiotherapies):ti,ab,kw OR (Neoadjuvant Chemoradiation Therapy):ti,ab,kw OR (Chemoradiation Therapy, Neoadjuvant):ti,ab,kw OR (Neoadjuvant Chemoradiation Therapies):ti,ab,kw OR (Therapy, Neoadjuvant Chemoradiation):ti,ab,kw OR (Neoadjuvant Chemoradiation Treatment):ti,ab,kw OR (Chemoradiation Treatment, Neoadjuvant):ti,ab,kw OR (Neoadjuvant Chemoradiation Treatments):ti,ab,kw OR (Treatment, Neoadjuvant Chemoradiation):ti,ab,kw OR (Neoadjuvant Chemoradiation):ti,ab,kw OR (Chemoradiation, Neoadjuvant):ti,ab,kw OR (Neoadjuvant Chemoradiations):ti,ab,kw

#6 #4 or #5

#7 Immunotherapy

#8 (Immunotherapies):ti,ab,kw

#9 #7 or #8

#10 #3 and #6 and #9

**Ovid**

#1 (Carcinoma, Non-Small-Cell Lung or Carcinoma, Non Small Cell Lung or Carcinomas, Non-Small-Cell Lung or Lung Carcinoma, Non-Small-Cell or Lung Carcinomas, Non-Small-Cell or Non-Small-Cell Lung Carcinomas or Non-Small-Cell Lung Carcinoma or Non Small Cell Lung Carcinoma or Carcinoma, Non-Small Cell Lung or Non-Small Cell Lung Carcinoma or Non-Small Cell Lung Cancer or Nonsmall Cell Lung Cancer or NSCLC or nsclc).ti,ab,kw.

#2 (Neoadjuvant Therapy or Neoadjuvant Therapies or Therapy, Neoadjuvant or Neoadjuvant Treatment or Neoadjuvant Treatments or Treatment, Neoadjuvant or Neoadjuvant Radiotherapy or Neoadjuvant Radiotherapies or Radiotherapy, Neoadjuvant or Neoadjuvant Radiation Treatment or Neoadjuvant Radiation Treatments or Radiation Treatment, Neoadjuvant or Treatment, Neoadjuvant Radiation or Neoadjuvant Radiation Therapy or Neoadjuvant Radiation Therapies or Radiation Therapy, Neoadjuvant or Therapy, Neoadjuvant Radiation or Neoadjuvant Radiation or Neoadjuvant Radiations or Radiation, Neoadjuvant or Neoadjuvant Systemic Therapy or Neoadjuvant Systemic Therapies or Systemic Therapy, Neoadjuvant or Therapy, Neoadjuvant Systemic or Neoadjuvant Systemic Treatment or Neoadjuvant Systemic Treatments or Systemic Treatment, Neoadjuvant or Treatment, Neoadjuvant Systemic or Neoadjuvant Chemotherapy or Chemotherapy, Neoadjuvant or Neoadjuvant Chemotherapies or Neoadjuvant Chemotherapy Treatment or Chemotherapy Treatment, Neoadjuvant or Neoadjuvant Chemotherapy Treatments or Treatment, Neoadjuvant Chemotherapy or Neoadjuvant Chemoradiotherapy or Chemoradiotherapy, Neoadjuvant or Neoadjuvant Chemoradiotherapies or Neoadjuvant Chemoradiation Therapy or Chemoradiation Therapy, Neoadjuvant or Neoadjuvant Chemoradiation Therapies or Therapy, Neoadjuvant Chemoradiation or Neoadjuvant Chemoradiation Treatment or Chemoradiation Treatment, Neoadjuvant or Neoadjuvant Chemoradiation Treatments or Treatment, Neoadjuvant Chemoradiation or Neoadjuvant Chemoradiation or Chemoradiation, Neoadjuvant or Neoadjuvant Chemoradiations).ti,ab,kw.

#3 (Immunotherapy or Immunotherapies).ti,ab,kw.

#4 #1 and #2 and #3

**Scopus**

(TITLE-ABS-KEY("Carcinoma, Non-Small-Cell Lung" OR "Carcinoma, Non Small Cell Lung" OR "Carcinomas, Non-Small-Cell Lung" OR "Lung Carcinoma, Non-Small-Cell" OR "Lung Carcinomas, Non-Small-Cell" OR "Non-Small-Cell Lung Carcinomas" OR "Non-Small-Cell Lung Carcinoma" OR "Non Small Cell Lung Carcinoma" OR "Carcinoma, Non-Small Cell Lung" OR "Non-Small Cell Lung Carcinoma" OR "Non-Small Cell Lung Cancer" OR "Nonsmall Cell Lung Cancer" OR "NSCLC" OR "nsclc") AND TITLE-ABS-KEY("Neoadjuvant Therapy" OR "Neoadjuvant Therapies " OR "Therapy, Neoadjuvant" OR "Neoadjuvant Treatment" OR "Neoadjuvant Treatments" OR "Treatment, Neoadjuvant" OR "Neoadjuvant Systemic Therapy" OR "Neoadjuvant Systemic Therapies" OR "Systemic Therapy, Neoadjuvant" OR "Therapy, Neoadjuvant Systemic" OR "Neoadjuvant Systemic Treatment" OR "Neoadjuvant Systemic Treatments" OR "Systemic Treatment, Neoadjuvant" OR "Treatment, Neoadjuvant Systemic" OR "Neoadjuvant Chemotherapy" OR "Chemotherapy, Neoadjuvant" OR "Neoadjuvant Chemotherapies" OR "Neoadjuvant Chemotherapy Treatment" OR "Chemotherapy Treatment, Neoadjuvant" OR "Neoadjuvant Chemotherapy Treatments" OR "Treatment, Neoadjuvant Chemotherapy" OR "Neoadjuvant Chemoradiotherapy" OR "Chemoradiotherapy, Neoadjuvant" OR "Neoadjuvant Chemoradiotherapies" OR "Neoadjuvant Chemoradiation Therapy" OR "Chemoradiation Therapy, Neoadjuvant" OR "Neoadjuvant Chemoradiation Therapies" OR "Therapy, Neoadjuvant Chemoradiation" OR "Chemoradiation Treatment, Neoadjuvant" OR "Neoadjuvant Chemoradiation Treatments" OR "Treatment, Neoadjuvant Chemoradiation" OR "Neoadjuvant Chemoradiation" OR "Chemoradiation, Neoadjuvant" OR "Neoadjuvant Chemoradiations") AND TITLE-ABS-KEY("Immunotherapy" OR "Immunotherapies"))

**ProQuest**

AB,TI("Carcinoma, Non-Small-Cell Lung" OR "Carcinoma, Non Small Cell Lung" OR "Carcinomas, Non-Small-Cell Lung" OR "Lung Carcinoma, Non-Small-Cell" OR "Lung Carcinomas, Non-Small-Cell" OR "Non-Small-Cell Lung Carcinomas" OR "Non-Small-Cell Lung Carcinoma" OR "Non Small Cell Lung Carcinoma" OR "Carcinoma, Non-Small Cell Lung" OR "Non-Small Cell Lung Carcinoma" OR "Non-Small Cell Lung Cancer" OR "Nonsmall Cell Lung Cancer" OR "NSCLC" OR "nsclc") AND AB,TI("Neoadjuvant Therapy" OR "Neoadjuvant Therapies " OR "Therapy, Neoadjuvant" OR "Neoadjuvant Treatment" OR "Neoadjuvant Treatments" OR "Treatment, Neoadjuvant" OR "Neoadjuvant Radiotherapy" OR "Neoadjuvant Radiotherapies" OR "Radiotherapy, Neoadjuvant" OR "Neoadjuvant Radiation Treatment" OR "Neoadjuvant Radiation Treatments" OR "Radiation Treatment, Neoadjuvant" OR "Treatment, Neoadjuvant Radiation" OR "Neoadjuvant Radiation Therapy" OR "Neoadjuvant Radiation Therapies" OR "Radiation Therapy, Neoadjuvant" OR "Therapy, Neoadjuvant Radiation" OR "Neoadjuvant Radiation" OR "Neoadjuvant Radiations" OR "Radiation, Neoadjuvant" OR "Neoadjuvant Systemic Therapy" OR "Neoadjuvant Systemic Therapies" OR "Systemic Therapy, Neoadjuvant" OR "Therapy, Neoadjuvant Systemic" OR "Neoadjuvant Systemic Treatment" OR "Neoadjuvant Systemic Treatments" OR "Systemic Treatment, Neoadjuvant" OR "Treatment, Neoadjuvant Systemic" OR "Neoadjuvant Chemotherapy" OR "Chemotherapy, Neoadjuvant" OR "Neoadjuvant Chemotherapies" OR "Neoadjuvant Chemotherapy Treatment" OR "Chemotherapy Treatment, Neoadjuvant" OR "Neoadjuvant Chemotherapy Treatments" OR "Treatment, Neoadjuvant Chemotherapy" OR "Neoadjuvant Chemoradiotherapy" OR "Chemoradiotherapy, Neoadjuvant" OR "Neoadjuvant Chemoradiotherapies" OR "Neoadjuvant Chemoradiation Therapy" OR "Chemoradiation Therapy, Neoadjuvant" OR "Neoadjuvant Chemoradiation Therapies" OR "Therapy, Neoadjuvant Chemoradiation" OR "Neoadjuvant Chemoradiation Treatment" OR "Chemoradiation Treatment, Neoadjuvant" OR "Neoadjuvant Chemoradiation Treatments" OR "Treatment, Neoadjuvant Chemoradiation" OR "Neoadjuvant Chemoradiation" OR "Chemoradiation, Neoadjuvant" OR "Neoadjuvant Chemoradiations") AND AB,TI("Immunotherapy" OR "Immunotherapies")

**Web of Science**

#4 3 AND #2 AND #1

#3 TS=(Immunotherapy or Immunotherapies)

#2 TS=(Neoadjuvant Therapy or Neoadjuvant Therapies or Therapy, Neoadjuvant or Neoadjuvant Treatment or Neoadjuvant Treatments or Treatment, Neoadjuvant or Neoadjuvant Radiotherapy or Neoadjuvant Radiotherapies or Radiotherapy, Neoadjuvant or Neoadjuvant Radiation Treatment or Neoadjuvant Radiation Treatments or Radiation Treatment, Neoadjuvant or Treatment, Neoadjuvant Radiation or Neoadjuvant Radiation Therapy or Neoadjuvant Radiation Therapies or Radiation Therapy, Neoadjuvant or Therapy, Neoadjuvant Radiation or Neoadjuvant Radiation or Neoadjuvant Radiations or Radiation, Neoadjuvant or Neoadjuvant Systemic Therapy or Neoadjuvant Systemic Therapies or Systemic Therapy, Neoadjuvant or Therapy, Neoadjuvant Systemic or Neoadjuvant Systemic Treatment or Neoadjuvant Systemic Treatments or Systemic Treatment, Neoadjuvant or Treatment, Neoadjuvant Systemic or Neoadjuvant Chemotherapy or Chemotherapy, Neoadjuvant or Neoadjuvant Chemotherapies or Neoadjuvant Chemotherapy Treatment or Chemotherapy Treatment, Neoadjuvant or Neoadjuvant Chemotherapy Treatments or Treatment, Neoadjuvant Chemotherapy or Neoadjuvant Chemoradiotherapy or Chemoradiotherapy, Neoadjuvant or Neoadjuvant Chemoradiotherapies or Neoadjuvant Chemoradiation Therapy or Chemoradiation Therapy, Neoadjuvant or Neoadjuvant Chemoradiation Therapies or Therapy, Neoadjuvant Chemoradiation or Neoadjuvant Chemoradiation Treatment or Chemoradiation Treatment, Neoadjuvant or Neoadjuvant Chemoradiation Treatments or Treatment, Neoadjuvant Chemoradiation or Neoadjuvant Chemoradiation or Chemoradiation, Neoadjuvant or Neoadjuvant Chemoradiations)

#1 TS=(Carcinoma, Non-Small-Cell Lung or Carcinoma, Non Small Cell Lung or Carcinomas, Non-Small-Cell Lung or Lung Carcinoma, Non-Small-Cell or Lung Carcinomas, Non-Small-Cell or Non-Small-Cell Lung Carcinomas or Non-Small-Cell Lung Carcinoma or Non Small Cell Lung Carcinoma or Carcinoma, Non-Small Cell Lung or Non-Small Cell Lung Carcinoma or Non-Small Cell Lung Cancer or Nonsmall Cell Lung Cancer or NSCLC or nsclc)
